# How Muscle Structure and Composition Influence Meat and Flesh Quality

**DOI:** 10.1155/2016/3182746

**Published:** 2016-02-28

**Authors:** Anne Listrat, Bénédicte Lebret, Isabelle Louveau, Thierry Astruc, Muriel Bonnet, Louis Lefaucheur, Brigitte Picard, Jérôme Bugeon

**Affiliations:** ^1^INRA, UMR1213 Herbivores, 63122 Saint-Genès-Champanelle, France; ^2^Clermont Université, VetAgro Sup, UMR1213 Herbivores, BP 10448, 63000 Clermont-Ferrand, France; ^3^INRA, UMR1348 PEGASE, 35590 Saint-Gilles, France; ^4^Agrocampus Ouest, UMR1348 PEGASE, 35000 Rennes, France; ^5^INRA, UR0370 QuaPA, 63122 Saint-Genès-Champanelle, France; ^6^INRA, UR1037 LPGP, Campus de Beaulieu, 35042 Rennes, France

## Abstract

Skeletal muscle consists of several tissues, such as muscle fibers and connective and adipose tissues. This review aims to describe the features of these various muscle components and their relationships with the technological, nutritional, and sensory properties of meat/flesh from different livestock and fish species. Thus, the contractile and metabolic types, size and number of muscle fibers, the content, composition and distribution of the connective tissue, and the content and lipid composition of intramuscular fat play a role in the determination of meat/flesh appearance, color, tenderness, juiciness, flavor, and technological value. Interestingly, the biochemical and structural characteristics of muscle fibers, intramuscular connective tissue, and intramuscular fat appear to play independent role, which suggests that the properties of these various muscle components can be independently modulated by genetics or environmental factors to achieve production efficiency and improve meat/flesh quality.

## 1. Introduction

The muscle mass of livestock and fish species used to produce human food represents 35 to 60% of their body weight. The striated skeletal muscles attached to the backbone are involved in voluntary movements and facilitate the locomotion and posture. Skeletal muscles exhibit a wide diversity of shapes, sizes, anatomical locations, and physiological functions. They are characterized by a composite appearance because in addition to muscle fibers, they contain connective, adipose, vascular, and nervous tissues. Muscle fibers, intramuscular connective tissue, and intramuscular fat play key roles in the determination of meat and fish flesh quality. Concerning meat and aquatic products, the different stakeholders, that is, producers, slaughterers, processors, distributors, and consumers, exhibit varied and specific requirements about quality that depend on their use of the products. Quality is generally described by 4 terms: security (hygienic quality), healthiness (nutritional quality), satisfaction (organoleptic quality), and serviceability (ease of use, ability to be processed, and prices). Satisfaction is driven by the qualities perceived by consumers. They include color, texture, and juiciness as well as flavor, which is associated with the aromas released in the mouth when the product is consumed. Satisfaction is also driven by technological qualities that reflect the ability of the product to be processed. They are mostly associated with a decrease in technological yield because of a decrease in water-holding capacity during cold storage (exudations) and cooking or because of damage that occurs after slicing. Better technological qualities are associated with low losses. The nutritional qualities depend primarily on the nutritional value of the fats, carbohydrates, and proteins that make up the food. A meat that is rich in proteins with a high proportion of essential amino acids and polyunsaturated fatty acids is considered to exhibit good nutritional quality. Finally, hygienic qualities reflect the product's capacity to be safely consumed. They are primarily related to the bacterial load of the product and the presence of chemical residues such as herbicides or pesticides and other environmental pollutants in the product. Among the cited qualities, critical points concerning the quality of beef for consumers are primarily tenderness, color, and healthiness. However, the primary cause of the consumer failure to repurchase beef is variability in tenderness [1]. In fish, the best quality is firm, cohesive flesh with a good water-holding capacity [2]. In meat and fish flesh, these qualities are influenced by many* in vivo* and* postmortem* (pm) factors such as species, genotypes, nutritional and environmental factors, slaughtering conditions, and pm processing. Because these factors also influence the structure and composition of skeletal muscle, their effect on meat quality could largely involve direct relationships between intramuscular biological properties and meat quality traits. However, such relationships are not always clear among species. Thus, the aim of this paper is to provide an overview of the structure and composition (muscle fibers, intramuscular connective tissue, and intramuscular fat) of muscle in livestock and fish and their relationships with the different qualities. Recent genomic studies on various rearing species to identify new biomarkers of meat quality have been previously reviewed [3] and when necessary will be briefly addressed in this paper.

## 2. Muscle Structure

### 2.1. Macroscopic Scale

Skeletal muscle consists of approximately 90% muscle fibers and 10% of connective and fat tissues. The connective tissue in skeletal muscle is divided into the endomysium, which surrounds each muscle fiber, the perimysium, which surrounds bundles of muscle fibers, and the epimysium, which surrounds the muscle as a whole [4, 5].

**Figure 1:**
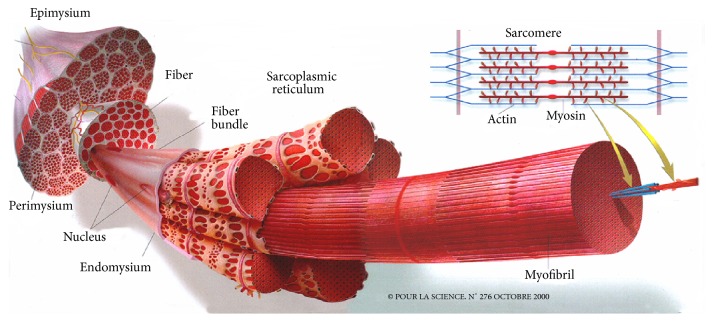
General organization of the muscle [9]. Skeletal muscle predominantly consists of muscle fibers and connective tissue. The latter is distributed on three levels of scale in the muscle: the endomysium, which surrounds each muscle fiber, the perimysium, which compartmentalizes muscle in fiber bundles, and finally the epimysium, which is the external envelope of muscle. Within the fibers, the myofibrils occupy nearly the entire intracellular volume. The contractile unit of the muscle fiber is the sarcomere.

**Figure 2:**
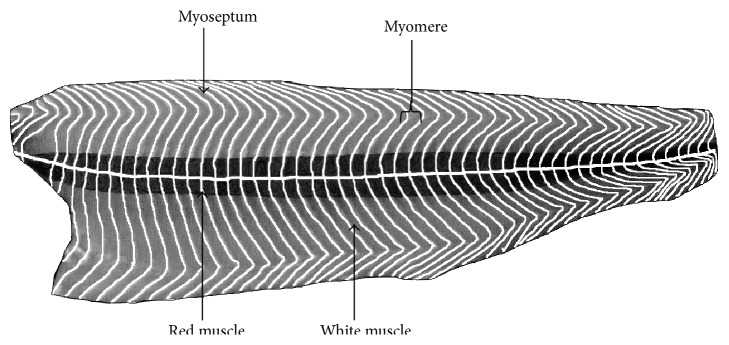
Diagram of a fish fillet (salmon) in longitudinal section, beneath the skin, to present the W-shape of myomere and the two muscle types.

**Figure 3:**
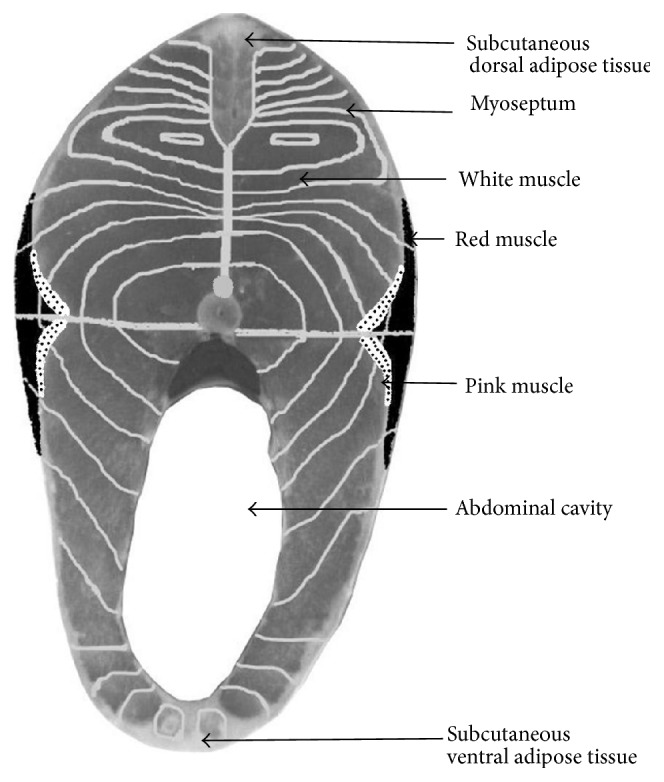
Diagrammatic organization and distribution of muscle mass on a trout cutlet (cross section).

When meat pieces consist of a unique muscle, the epimysium is removed. However, when a meat piece includes several muscles, only the external epimysium is absent (Figure 1). Skeletal muscle also contains fat tissue and to a lesser extent vascular and nervous tissues. In fish, the edible part, the fillets, consists of several muscles (myomeres), which are fitted into one another and separated by connective tissue sheaths of a few millimeters thickness, known as myosepta. The myosepta exhibit structural continuity from the vertebral axis to the skin. Their role is to ensure the transmission of the fiber-contraction forces of one myomere to another and to the skeleton and skin. This particular structure, with alternating muscle and connective sheaths, is termed a metameric organization. In a “round” fish of commercial size, the shape of the myomeres of a fillet resembles a W (Figure 2). However, this organization is more complex in cross section (i.e., a cutlet) (Figure 3). The myosepta can be considered to be the epimysia of terrestrial livestock species muscle. The other intramuscular connective tissues of fish exhibit a similar organization to that found in terrestrial animals. A unique characteristic of fish muscle is an anatomical separation at the macroscopic scale of the three main types of muscle: a major white muscle, a superficial red muscle (along the skin), and an intermediate pink muscle. These muscles are present in each myomere (Figure 3). The fish fillet also contains intramuscular adipose tissue located within a myomere between the myofibers and in the perimysium, but mainly in the myosepta separating myomeres.

### 2.2. Microscopic Scale

**Figure 4:**
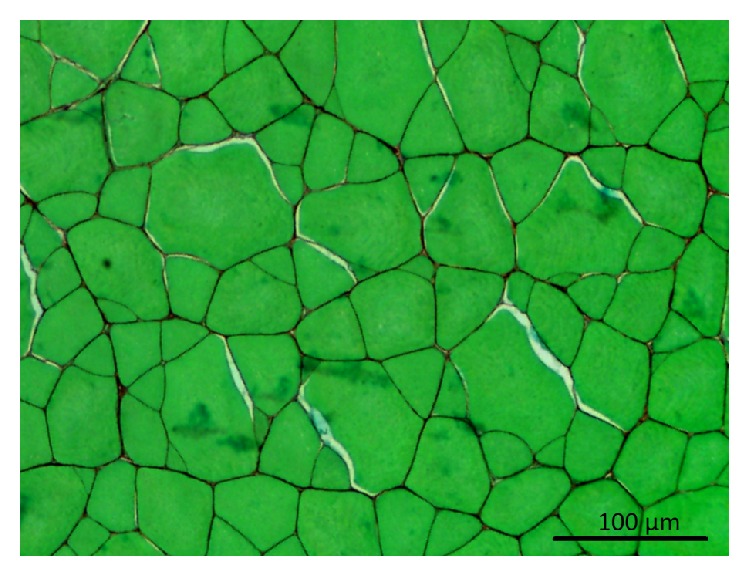
Histologic cross section of Sea Bass (*Dicentrarchus labrax*) white muscle stained with sirius red and fast green. Muscle consists of large and small fibers (approximately 100 and 10 microns in diameter, resp.).

Muscle fibers are elongated, multinucleated, and spindle-shaped cells of approximately 10 to 100 micrometers diameter and a length that ranges from a few millimeters in fish to several centimeters in terrestrial animals. In all species, the fiber size increases with animal age and is an important parameter of postnatal muscle growth. Muscle fiber plasma membrane is known as the sarcolemma. The cross-sectional area (CSA) of fibers depends on their metabolic and contractile types (see Section 3.1 for the types of muscle fiber). In fish, the fiber size distribution varies according to the importance of the hypertrophic (increase in cell size due to an increase in volume) and the hyperplasic growth stages (an increase of muscle volume due to an increase in cell number). The simultaneous presence of small and large fibers results in the so-called “mosaic” structure typically encountered in fish (Figure 4).

**Figure 5:**
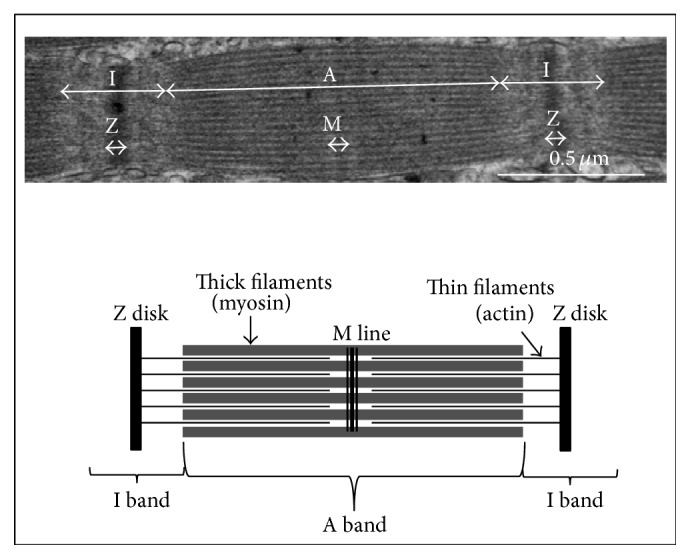
The sarcomere, which is the smallest contractile unit of the muscle, is delimited by the Z disks. It consists of at least thirty different proteins, of which the most abundant are myosin and actin.

Regardless of the species, the myofibrils lined up in bundles occupy nearly the entire intracellular volume of muscle fibers. Myofibrils have a diameter of approximately 1 *μ*m and consist of small subunits: the myofilaments (Figure 1). Longitudinal cross sections of myofibrils observed by electron microscopy exhibit alternating dark (A bands) and light areas (I bands). Each I band is divided into two portions by a Z line. The repeating unit found between two Z lines is the sarcomere, which is the contractile functional unit of the myofibril (Figure 5). Thin myofilaments primarily consist of actin, the troponins T, I, and C (which regulate muscle contraction) and tropomyosin arranged end to end along the actin filament. Thick myofilaments primarily consist of an assembly of myosin molecules whose ATPase activity catalyzes the breakdown of adenosine triphosphate (ATP) into adenosine diphosphate (ADP) and provide the chemical energy required for muscle contraction. Sarcoplasm, that is, the cytoplasm of muscle fibers, contains many soluble proteins, including enzymes of the glycolytic pathway and myoglobin, which carries oxygen to the mitochondria and stains cells red. It also contains glycogen granules, which represent the primary local energy reserve of muscle cells, in addition to lipid droplets.

## 3. Muscle Biochemical Composition

Skeletal muscles contain approximately 75% water, 20% protein, 1–10% fat, and 1% glycogen. The biochemical properties of the major muscle components (i.e., myofibers, connective tissue, and adipose tissue) are described in the following.

### 3.1. Muscle Fibers

Muscle fibers are generally characterized by their contractile and metabolic properties [6, 7]. The contractile properties primarily depend on myosin heavy-chain isoforms (MyHCs) present within the thick filaments. In most mature mammalian skeletal striated muscles, four types of MyHC are expressed: I, IIa, IIx, and IIb. The ATPase activity of these MyHCs is related to the speed of contraction: slow (type I) and fast (types IIa, IIx, and IIb). Type I fibers exhibit low-intensity contractions but are resistant to fatigue. They predominate in postural and respiratory muscles. Muscle contraction requires energy from ATP, whose requirements differ widely among the muscle fiber types [8].

Two major pathways of ATP regeneration are used in the muscle: the oxidative (aerobic) pathway through which pyruvate is oxidized by the mitochondria, and the glycolytic (anaerobic) pathway wherein pyruvate is converted into lactic acid in the sarcoplasm. The relative importance of these two pathways determines the metabolic fiber type: oxidative (red; rich in myoglobin which is the oxygen carrier and pigment responsible for the red color), or glycolytic (white; nearly devoid of myoglobin because oxygen requirements are highly limited). Generally, oxidative red fibers exhibit a smaller CSA than glycolytic white fibers. However, the differential size between fiber types can vary depending on the muscle and within the same muscle. For example, oxidative fiber CSA is greater than glycolytic fiber CSA in the red part of the* semitendinosus* muscle in pigs [10]. Similarly in the* Rectus abdominis* muscle of cattle, the oxidative red fiber CSA is larger than white glycolytic fiber CSA [11]. Finally, muscle fibers are dynamic structures that can switch from one type to another one according to the following pathway: I↔IIA↔IIX↔IIB [12]. A summary of the different fiber type properties in mature mammalian skeletal muscle is shown in Table 1. Despite the obvious presence of their genes, none of the three isoforms of adult fast MyHC are present in the mature muscles of all mammalian species. In fact, the IIb MyHC is not expressed in sheep and horses and has been found only in certain cattle muscles with strong differences between breeds [13]. In contrast, strong expression of IIb MyHC is observed in skeletal muscles of conventional pig breeds selected for leanness and high growth performance [14]. Regardless of the species, the most important factor that determines muscle fiber composition is muscle type, likely in relation to its specific physiological function. For a given muscle, the fiber composition varies depending on the species. Thus, pig* Longissimus* muscle contains approximately 10% type I fibers, 10% IIA, 25% IIX, and 55% IIB, whereas bovine* Longissimus* contains on average 30% type I fibers, 18% IIA, and 52% IIX. The composition of muscle fibers is also influenced by breed, gender, age, physical activity, environmental temperature, and feeding practices. As in mammals, the muscle fibers of birds can be classified based on their contractile and metabolic activities. However, additional classes, for example, the multitonic innervated slow fibers of types IIIa and IIIb, which are specific to avian muscles, have been described [15]. In birds, it is difficult to match an isoform of MyHC with a fiber type due to the simultaneous presence of adult and developmental types of MyHC in mature fibers. Fish also exhibit different types of muscle fiber characterized by their contractile and metabolic properties. However, in contrast to mammals or birds, an anatomical separation between the two main fiber types can be observed in fish. For example, in trout, fast fibers (similar to mammalian IIB fibers) are found in the center in a cross-sectional body area, and slow fibers (similar to the mammalian type I) are found at the periphery along a longitudinal line under the skin [16]. In addition to these two main fiber types, minor types, such as the intermediate type (e.g., the pink fiber type, comparable to the type IIA) can be found in certain species or at certain stages of development. The two main types of white and red fiber have been associated with the expression of fast and slow MyHC, respectively [17]. However, it can be difficult to systematically match a MyHC isoform with a fiber type due to the simultaneous presence of several MyHCs within the same fiber in fish, particularly in the small muscle fibers.

### 3.2. Intramuscular Connective Tissue

The connective tissue that surrounds muscle fibers and fiber bundles is a loose connective tissue. It consists of cells and an extracellular matrix (ECM) that primarily consists of a composite network of collagen fibers wrapped in a matrix of proteoglycans (PGs) [4, 18, 19]. This paper focuses on the molecules that have been demonstrated or suspected to play a role in the determination of meat sensory quality. The collagens are a family of fibrous proteins. Regardless of the collagen type, the basic structural unit of collagen (tropocollagen) is a helical structure that consists of three polypeptide chains coiled around one another to form a spiral. Tropocollagen molecules are stabilized by interchain bonds to form fibrils of 50 nm diameter. These fibrils are stabilized by intramolecular bonds (disulphide or hydrogen bridges) or intermolecular bonds (including pyridinoline and deoxypyridinoline), known as cross-links (CLs). Various types of collagen are found in skeletal muscle. Fibrillar collagens I and III are the major ones that appear in mammals [19]. In fish, collagen types I and V predominate [20]. The other main components of connective tissue are the PGs [21]. The PGs are complex multifunctional molecules that consist of a core protein of molecular weight that ranges from 40 to 350 kDa, linked by covalent bonds to several dozen glycosaminoglycan chains (GAGs). PGs form large complexes by binding to other PGs and to fibrous proteins (such as collagen). They bind cations (e.g., sodium, potassium, and calcium) and water [22]. The proportion and the degree of intramuscular collagen cross-linking depend on muscle type, species, genotype, age, sex, and level of physical exercise [23]. The total collagen content varies from 1 to 15% of the muscle dry weight in adult cattle [19], whereas it varies between 1.3 (*Psoas major*) and 3.3% (*Latissimus dorsi*) of muscle dry weight in Large White pigs at the commercial slaughter stage [24]. In poultry, the collagen represents 0.75 to 2% of the muscle dry weight [25]. In fish, variable contents have been reported according to species (quantities vary from 1 to 10% between sardines and congers [26]), within species and between the front and caudal parts (richer) of the fillet [27]. PGs represent a small proportion of the muscle dry weight (0.05% to 0.5% in cattle according to muscles) [28].

### 3.3. Intramuscular Fat

In mammals, reserve fat is located in several external and internal anatomical locations such as around and within the muscle for the intermuscular and intramuscular (IMF) fats. In this paper, we focus essentially on IMF because intermuscular fat is trimmed during cutting and thus has less impact on pork and beef meat. In fish, fat are located subcutaneously and within the perimysium and myosepta, and mainly the latter contribute to flesh quality and is considered in this paper. IMF mostly consists of structural lipids, phospholipids, and storage lipids (the triglycerides). The latter are primarily (approximately 80%) stored in the muscle adipocytes found between fibers and fiber bundles, and a minor proportion (5–20%) is stored as lipid droplets within myofibers in the cytoplasm (intracellular lipids) [29]. Between muscle types, the phospholipid content is relatively constant (i.e., ranging from 0.5 to 1% of fresh muscle in pigs), whereas the muscle triglyceride content is highly variable whatever the species [30, 31]. The IMF content strongly depends on the size and number of intramuscular adipocytes. In pigs [32, 33] and cattle [30, 34], the interindividual variation in IMF content of a given muscle between animals of similar genetic background has been associated with variation in the number of intramuscular adipocytes. In contrast, variation in the IMF content of a given muscle between animals of the same genetic origin and subjected to different dietary energy intakes has been demonstrated to be associated with variation in adipocyte size [33]. In fish, the increase in myosepta width is likely related to an increase in the number and size of adipocytes [35]. The IMF content varies according to anatomical muscle origin, age, breed, genotype, diet, and the rearing conditions of livestock [30, 36–39]. For example, Chinese and American pigs (e.g., Meishan and Duroc, resp.) or European local pig breeds (e.g., Iberian and Basque) have higher levels of IMF than do European conventional genotypes, such as Large White, Landrace, or Pietrain [40]. The IMF content varies from 1 to approximately 6% of the fresh* Longissimus* muscle weight in conventional genotypes of pigs at the commercial slaughter stage, with values up to 10% in certain breeds [38]. In cattle, the IMF content of* Longissimus* muscle varies from 0.6% in Belgian Blue to 23.3% in Black Japanese at slaughter at 24 months of age [41]. In French cattle breeds, it has been demonstrated that selection on muscle mass has been associated with a decrease in IMF and collagen contents. For example, the main meat breeds Charolaise, Limousine, and Blonde d'Aquitaine have less IMF than hardy breeds, such as Aubrac and Salers, all exhibiting lower IMF levels than dairy breeds [42] or American or Asian breeds reared under the same conditions [36, 43]. In fish, the IMF content also varies between species from less than 3% in “lean” species such as cod to more than 10% in “fatty” species, such as Atlantic salmon [37], but also within species. For example, in salmon flesh, fat content may vary between 8 and 24% [44].

## 4. Relations between the Different Muscle Components

**Figure 6:**
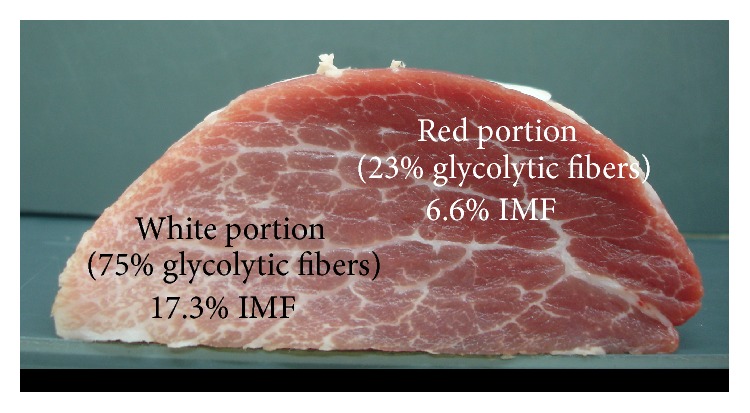
*Semitendinosus* muscle cross section from a Basque pig at 145 kg live weight. The intramuscular fat content (IMF) is approximately three times higher in the white glycolytic than in the red oxidative portion of the muscle (Lefaucheur, personal communication).

Studies based on comparisons between muscle types indicate that IMF content is typically positively correlated with the percentage of oxidative fibers and negatively with the glycolytic fibers [45]. Although oxidative fibers, particularly slow fibers, exhibit a higher intramyocellular lipid content than fast glycolytic fibers do [46] and although the IMF content has often been found to be higher in oxidative than in glycolytic pig muscles (i.e.,* Semispinalis* versus* Longissimus* muscles) [47], many studies also indicate no strict relationship between total IMF content and muscle fiber composition [6]. In extreme cases, the IMF content can be three times higher in the white glycolytic than in the red oxidative part of the* Semitendinosus* muscle in the pig [34] (Figure 6). A negative correlation between IMF content and the oxidative metabolism was also found in the pig* Longissimus* muscle in a functional genomic approach [48]. However, positive genetic and phenotypic correlations were observed between IMF content and muscle fiber CSA in pig* Longissimus* muscle [49]. In fish, in which white and red muscles are anatomically separated, it is assumed that red muscles exhibit more elevated fat content than white muscles due to higher numbers of fat cells in the perimysium and higher numbers of lipid droplets within muscle fibers. In Atlantic salmon, a negative genetic correlation (rg = −0.85) has been reported between the total number of fibers and the IMF content, which suggests that, at a similar weight, selection for low IMF would result in an increase in the number of fibers [50]. Additionally, a negative correlation between collagen content and IMF (rg = −0.8) has been observed, which indicates that an increase in IMF would cause a relative decrease in muscle collagen content likely due to its “dilution” within muscle tissue [51]. No systematic relationship between the biochemical characteristics of the connective tissue and muscle fiber type has been found in meat-producing animals. In contrast, in fish, collagen content is higher in red than in white muscles [52].

## 5. Mechanisms of Muscle pm Changes and Quality of Meat and Flesh: Modulation by Muscle Properties

**Figure 7:**
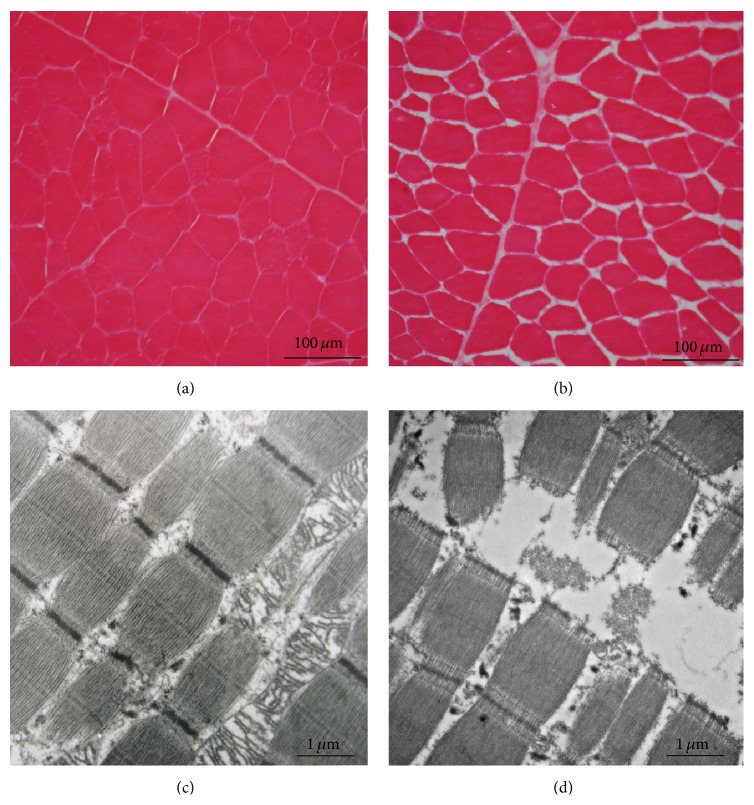
(a, b) Histological cross sections of bovine semitendinosus muscle taken at slaughter (a)* and 12 days postmortem.* (b) Observed by light microscopy. During storage (4°C in a cold room), cells shrink and extracellular spaces increase. (c, d) Histological longitudinal section of bovine* semitendinosus* muscle taken at slaughter (c)* and 12 days postmortem* (d) observed by transmission electron microscopy. At the ultrastructural scale, proteolytic action of enzymes causes breaking of myofibrils along the Z disks.

After slaughter, the meat is typically stored in a cold room at 4°C for 2 to 30 days depending on species, subsequent processing methods, and packaging. The longest storage periods are used for beef (one to two weeks for carcasses to one month for meat pieces stored under vacuum) to facilitate a natural tenderizing (aging) process. The reduction of muscle fiber CSA observed during the refrigeration results from a lateral shrinkage of myofibrils whose amplitude depends on the slaughter stress of animals and of the stunning technology (Figure 7) [53]. The aging phase is characterized by various ultrastructural changes and results in the fragmentation of muscle fibers. The action of different proteolytic systems results in characteristic myofibrillar ruptures along the Z lines (Figure 7). Mitochondria are deformed and their membranes altered [18, 54]. As a consequence of the degradation of costameres, that is, the junction of cytoskeletal proteins to the sarcolemma, the sarcolemma separates from peripheral myofibrils [55]. According to Ouali et al. [54], the enzymatic process starts as soon as bleeding occurs, with an activation of caspases, which are responsible for damage to cellular components during apoptosis. Other proteolytic systems (e.g., calpain, proteasome, and cathepsins) take over to continue the protein degradation of cells and muscle tissue [56].

Connective tissue also undergoes morphological changes during meat-aging [19, 21], which are detectable as early as 12 h pm in chickens [25] but only after 2 weeks pm in cattle [57]. This degradation facilitates the solubilization of collagen during cooking, thus improving the tenderness of cooked meat. An indirect effect of PGs on the tenderness of cooked meat has also been suggested. In fact, during aging, reduction of the perimysium resistance is associated with decreasing amounts of PGs along with an increase in collagen solubility due to the increased activity of certain enzymes. One hypothesis is that PGs may be degraded (spontaneously or enzymatically) during maturation and no longer protect collagen from enzymatic attacks [21]. In fish, flesh tenderization is associated with a gradual breakdown of the endomysium [58] and a detachment of the fibers from one another due to the rupture of ties with the endomysium and with the myosepta [59]. Soft-flesh fish demonstrate more endomysium (collagen, PGs) breakdown [60]. Fish myofibrils exhibit weak ultrastructural changes of the actomyosin complex, unlike bovine muscle [61]. Thus, in sea bream (*Sparus aurata*), I and Z bands are only partially degraded after 12 days of refrigerated storage [62].

## 6. Relations between Muscle Properties and Meat Quality

Among the various components of meat quality, the technological, nutritional, and sensory dimensions are considered. The nutritional quality component is primarily determined by the chemical composition of muscle tissue at slaughter, whereas the technological and sensorial components result from complex interactions among the chemical composition and metabolic properties of the muscle at slaughter and pm biochemical changes that lead to its conversion into meat [56, 63]. The structure and muscle composition, the kinetics of pm changes, and the additional meat use and processing methods that are applied (e.g., mincing, cooking) vary according to species and cuts, which results in major intrinsic differences in meat qualities between animal species and cuts. Therefore, the hierarchy between the most desired qualitative components varies between species. Prominent examples include tenderness in cattle, firmness in fish flesh, and water-holding capacity in pigs and chickens.

### 6.1. Technological Quality

After slaughter, depending on the species and the markets, the carcasses are stored in a cold room and then cut into pieces or muscles. During storage, the internal structure of muscles changes. The muscle fibers shrink laterally while expelling intracellular water to extracellular spaces, whose size increases. Subsequently, this water is expelled at the cut ends of muscles [53]. Regarding processing into cooked products, the technological quality is related to the water-holding capacity of meat, that is, its ability to retain its intrinsic water. The water-holding capacity is strongly influenced by the rate and extent of decrease in the pm pH. A high rate combined with a high muscle temperature (e.g., from stress or intensive physical activity directly prior to slaughter) causes denaturation of muscle proteins, reduced water-holding capacity and increase exudation, and cooking loss of meat in pigs and poultry. A large extent of pH decrease (i.e., acid meat) reduces the net electric charge of proteins, which also reduces the water-holding capacity [64, 65]. Measuring pH within one hour after slaughter and then on the following day to assess the rate and extent of pH decline, the determination of color and water loss during cold storage are the main indicators of the technological quality of meat. Muscle fiber composition influences the technological quality of meat, such as the water-holding capacity, which depends on the evolution of muscle pm pH kinetics and temperature. The pm pH decrease generally occurs faster in glycolytic muscles than in oxidative ones [66] although this relationship is not systematic. In fact, the pH at 45 min pm is much lower in pork* Psoas major* muscle (27% fiber I) than in* Longissimus* muscle (10% I fibers) [6], which could be explained by the lower buffering capacity of type I fiber (Table 1) or differences in the kinetics of pm temperature decline according to the anatomical location of muscles. In addition, stimulation of muscle glycolytic metabolism in the hour following slaughter increases the rate of pH decrease, which when combined with a high muscle temperature may result in protein denaturation and pale, soft, and exudative (PSE) syndrome in white muscles, particularly in pigs and chickens. In contrast, the extent of pm pH drop (ultimate pH; typically determined 24 h pm) is consistently greater in white glycolytic than in red oxidative muscles due to a higher muscle glycogen content* in vivo* and during slaughter in the fast-twitch white glycolytic fibers. In Large White pig* Longissimus* muscle, the increase in rate and extent of pm pH decrease are associated with a paler color and higher luminance and exudation [49, 67]. In pigs, two major genes that substantially influence the kinetics of pm pH decrease and water-holding capacity have been identified. Mutation in the RYR1 gene (also known as the halothane gene), which encodes a ryanodine receptor that is part of the calcium release channel of the sarcoplasmic reticulum, is responsible for a rapid decrease in pm pH and the development of PSE meat [68]. Another pork quality defect is due to a mutation in the PRKAG3 gene that encodes a subunit of the AMP-activated protein kinase (AMPK) [69]. This mutation results in a very high muscle glycogen level at slaughter (+70%), particularly in the glycolytic muscles, which is responsible to a significant extent for the pm pH decrease and “acid meat” with low water-holding capacity. Interestingly, the Longissimus muscle of mutated PRKAG3 pigs contains more oxidative fibers [47] and a lower buffering capacity [70] which contributes to the low ultimate pH in addition to the greater lactate production from glycogen. A recent proteomic study in cattle revealed some correlations between metabolic, antioxidant and proteolytic enzymes with pH decline. These data allow a better understanding of the early pm biological mechanisms involved in pH decline [71].

### 6.2. Nutritional Quality

Meat and flesh are an important source of proteins, essential amino acids (AAs), essential fatty acids (FAs), minerals, and vitamins (A, E, and B), which determine nutritional quality. The AA profile is relatively constant between muscles or between species [72]. However, collagen-rich muscles have a lower nutritional value because of their high glycine content, a nonessential AA [19]. Compared with white muscles, red muscles have larger myoglobin content and thereby provide higher amounts of heme iron, which is easily assimilated by the body. Although IMF constitutes a small fraction of muscle mass, it is involved in human FA intake because the content and nature (i.e., the profile) of meat FA varies according to species, the anatomical origin of a given muscle, and animal diet [30, 73]. Dietary strategies have been intensively studied and optimized to decrease saturated fatty acid intakes and increase cis-monounsaturated and polyunsaturated fatty acids or other bioactive lipids in animal-derived products for human consumption [30, 73]. In addition, because n-3 fatty acids with more than 20 carbons are primarily incorporated into phospholipids rather than into triglycerides, it is possible to enrich meat content in these polyunsaturated fatty acids without increasing IMF. For example, regarding bioactive lipids, the peculiarity of meat from ruminants is the presence of fatty acids that directly or indirectly result from ruminal biohydrogenation and that are proposed to be bioactive fatty acids, such as rumenic acid, which is the main natural isomer of the conjugated linoleic acids [30] and known to prevent certain forms of cancer in animal models. However, during pm aging and meat storage, lipids undergo alterations (e.g., peroxidation), whose importance depends on the FA composition of the meat. These alterations may impair the sensory (e.g., color, flavor) and nutritional qualities of the meat [63, 74].

### 6.3. Sensory Quality

#### 6.3.1. Color and Appearance

The composition of muscle fibers influences meat color* via* the amount and the chemical state of myoglobin. The high myoglobin content of type I and type IIA fibers results in a positive relationship between the proportion of these fibers and red color intensity. In deep muscles and meat stored under vacuum, myoglobin is in a reduced state and exhibits purple red color. When exposed to oxygen, myoglobin is oxygenated into oxymyoglobin, which gives the meat an attractive bright red color. During meat storage, myoglobin can be oxidized into metmyoglobin, which produces a brown, unattractive color that is negatively perceived by consumers [75, 76]. Many* ante-* and pm factors, such as animal species, sex, age, the anatomical location and physiological function of muscles, physical activity, the kinetics of pm pH decrease, the carcass chilling rate, and meat packaging, influence the concentration and chemical state of pigments and consequently meat color [77]. Muscles from cattle, sheep, horses, and migratory birds (e.g., geese, ducks) that contain high proportions of type I fibers rich in myoglobin are thus prone to metmyoglobin formation and decreased color stability. In contrast, a high proportion of glycolytic fibers results in the production of white meat, as found in chickens and pigs. Double-muscled cattle (mutation in the* myostatin* gene) present muscles with a high proportion of fast glycolytic fibers and consequently pale meat [3].

Meat color also depends on diet. For example, the feeding of calves with cow's milk that is free of iron limits myoglobin biosynthesis, which results in pale meat as a result of iron deficiency.

In fish, only the superficial lateral red muscle, which is rich in myoglobin, exhibits intense (generally brown) color, whereas the white muscle is rather translucent. In the case of salmonids, the orange-red color of the flesh is due to the presence of food-supplied carotenoid pigments, such as astaxanthin, in the muscle fibers. Differences in lipid levels can result in variations in the thickness of myosepta (i.e., the "white stripes" trait), which can be detected by a trained sensory panel in fish that exhibit the contrasted muscle yields associated with different lipid contents [78]. On a given fish slice (cross section), red muscles can also be observed on the edge of white muscle, which represents approximately 90% of the muscle. Consumer perception of the red muscle, which oxidizes quickly pm to brown and then to black, is generally negative, and this red muscle is occasionally removed for premium products (e.g., smoked fillets). In addition to color, the quantity and distribution of marbling within a muscle slice affect appearance and thus can affect the acceptance of meat and meat products by consumers (cf.  Section 6.3.3). In fish, another major defect of flesh (fillet) appearance is the so-called “gaping” defect, which results from the partial disruption of the myosepta or the fiber/myosepta interface. The biological and/or technological origin of this quality defect remains unclear.

#### 6.3.2. Tenderness

Tenderness and its variability are the most important sensory characteristic for beef consumers. Beef meat has a much higher basic toughness (determined by the proportion, distribution, and nature of the intramuscular connective tissue) and lower pm tenderization process than those of pork or poultry [63]. Thus, the pm aging duration is essential for beef tenderness [79]. In pigs and poultry, the pm acidification kinetics of muscles, which is faster than in cattle [79], strongly influences the texture (i.e., juiciness, tenderness) and the technological properties of meat (e.g., water-holding capacity) [63]. In cattle, the relationships between fiber characteristics and tenderness are complex and vary according to muscle, sex, age, and breed [80]. For example, among bulls,* Longissimus thoracis* tenderness is often associated with a decrease in fiber CSA and an increase in oxidative metabolism, whereas in the* Vastus lateralis* and* semitendinosus* muscles, the higher that the glycolytic activity is, the tenderer the meat is [81]. However, a negative correlation between the intensity of the oxidative metabolism and tenderness has also been observed in the* Longissimus* muscle of cattle [82]. Using biomarkers of beef tenderness Picard et al. [83] demonstrated that in breeds characterized by a muscle metabolism more fast glycolytic, such as the French beef breeds, the most tender* Longissimus thoracis* are the most oxidative. On the contrary, in breeds whose muscle metabolism is more oxidative, such as Aberdeen Angus, the most glycolytic* Longissimus thoracis* are the tenderest. This is in accordance with the fact that in breeds that exhibit oxidative muscles, such as Angus or dairy breeds, rib steaks with low red color intensity are tenderer. In contrast, among the main French beef breeds that exhibit more glycolytic muscles, the reddest the muscle is, the tenderer the meat is [83]. A higher proportion of glycolytic fibers could improve the tenderness of certain muscles by accelerating pm aging due to the presence of a higher calpain/calpastatin ratio (two proteins involved in proteolysis) [84] in the meat of animal species with slow meat-aging, such as cattle and sheep [82]. However, for other authors, the improvement in meat tenderness associated with the increase in the type I fiber proportion is explained by the higher protein turnover and associated proteolytic activity in the oxidative fibers [85]. Among bulls, except for rib steak, meat tenderness does not seem to be associated with fiber CSA but with the metabolic properties of muscle fibers.

In pigs, a functional genomic study has reported a negative impact of the abundance of fast fibers and of high glycolytic metabolism on meat tenderness [48]. This study also demonstrates that reduced expressions of protein synthesis genes (e.g., antiapoptotic heat shock-proteins genes and the calpastatin gene) and an increase in the expression level of genes involved in protein degradation (particularly proteasomes) are associated with a lower shear force (i.e., improved tenderness) at 1 day pm. A negative relationship between average fast glycolytic fiber CSA and tenderness has been demonstrated in pigs [86]. Therefore, a strategy aimed at increasing the total number of fibers combined with moderate fiber CSA and an increase in the percentage of slow-twitch oxidative fibers could be a promising means to increase muscle quantity while preserving the sensory quality of pork [6]. In contrast, in chickens, an increase in fiber CSA in the* Pectoralis* muscle is associated with a decrease in muscle glycogen content, higher ultimate pH and water-holding capacity, and improved tenderness [87]. However, contradictory data for chickens also report negative effects of fiber CSA on meat water-holding capacity and tenderness [88]. In fish, comparisons between species have observed a negative correlation between the mean diameter of muscle fibers and flesh firmness. However, this relationship seems more controversial within species: similar results have been found for smoked Atlantic salmon and the raw flesh of brown and rainbow trout, whereas other studies did not demonstrate a relationship between fiber size and the texture of salmon or cod flesh. Altogether, as in pigs, it appears that hyperplasic rather than hypertrophic muscle growth is better for the quality of fish products.

Connective tissue influences meat tenderness by its composition and structure [4], particularly in cattle, whereby collagen is generally considered to be the major determinant of the shear force. However, there are substantial differences between raw and cooked meat. The shear force of raw meat is highly correlated with its collagen content [21, 89]. In cooked meat, the level of correlation between the content, thermal solubility, or cross-linking level of collagen and meat shear force is unclear and varies according to muscle type and cooking conditions [90, 91]. During heating, the collagen fibers shrink and pressurize muscle fibers with a magnitude that depends on the degree of collagen cross-linking and the organization of the endomysium and the perimysium. The level of interaction between collagen and muscle fibers modulates the thermal denaturation of collagen (i.e., its gelatinization) and therefore the development of meat tenderness during cooking [89]. In pigs and chickens, it is generally considered that collagen has a limited impact on meat sensory quality. The reason is that the animals are slaughtered at a relatively early physiological stage, at which intramuscular collagen is not significantly cross-linked [19].

In addition to its composition, the structure of connective tissue, in particular its organization and the size of the perimysium bundles (which determine the grain of the meat, particularly in beef), also plays a role in the development of meat texture [92]. According to Purslow [23], the relationships between the grain of meat and texture indicate that tenderness is positively correlated with the proportion of small diameter bundles (termed primary bundles) but that this parameter does not accurately predict tenderness. Ellies-Oury et al. [80] demonstrated no significant relationship between grain of meat and tenderness evaluated by a trained sensory panel, shear force, or collagen content and solubility. Additionally, the shear force of the muscle increases with the thickness of the secondary perimysium bundles in cattle [93] and pigs [94]. Larger bundles (e.g., tertiary, quaternary) occur but are rarely considered in studies that address meat tenderness. Thus, their influence on the structure of muscle connective tissue and meat tenderness remains unclear.

In fish, comparisons among species have demonstrated a positive relationship between the firmness of raw flesh and its collagen content. However, this relationship was not observed within species. Regarding the influence of collagen cross-linking on the firmness of raw flesh, only a low relationship (*R* ^2^ = 0.25) between the content in hydroxylysyl pyridinoline (CLs) and the mechanical strength of the fillet has been observed in salmon [95]. Because of its low thermal stability compared with that of mammals, muscle fish collagen does not maintain its structural properties during cooking. Thus, the texture of the cooked flesh mostly depends on the myofibrillar proteins. Comparisons between species have noted positive correlations between muscle collagen content and the tenderness and elasticity of the cooked flesh [26]. However, none of these results were found within fish species. Fish species with firm flesh exhibit a highly dense network of collagen fibers in the endomysium, whereas this network is much looser in the less firm flesh species [96].

#### 6.3.3. Juiciness and Flavor

In cattle and lambs, an increased proportion of type I fibers is associated with improved meat juiciness and flavor [85, 97]. This favorable effect on flavor is probably explained by the high phospholipid content of type I fibers, the phospholipids being a major determinant of the flavor of cooked meat [98]. However, the high content of polyunsaturated FAs in phospholipids increases the risk of a rancid taste. In pigs, a high percentage of fast oxidoglycolytic fibers impairs the water-holding capacity and juiciness of the meat [85, 99]. IMF is often recognized as playing a key role in the determination of sensory qualities of meat or flesh in different animal species by positively influencing juiciness, flavor, and tenderness, although its influence on sensory traits varies among species [37]. It is generally accepted that very low levels of IMF result in dry meat with low taste. However, a high correlation between IMF and sensory quality ratings assigned by a trained panel may be observed only when important variations and high maximal levels of IMF occur (i.e., in pigs) [100]. In fact, other factors can modulate this relationship, such as the ultimate pH of meat in pigs, or the content and the type of intramolecular CLs of collagen in cattle [37]. For example, beef with similar levels of IMF (approximately 3.2%) but issued from four different breeds (Angus, Simmental, Charolais, and Limousine) exhibited similar flavor but higher juiciness in the Limousine and lower juiciness in the Angus breeds [101]. Regarding the assessment of fresh meat and meat products by consumers, the influence of IMF seems contradictory. Before consumption, consumers prefer less marbled pork, whereas at the time of consumption, the most marbled meats are considered to be juicier, tenderer, and tastier [100, 102, 103]. Although fats are a key factor in the development of flavor during meat cooking and in meat juiciness, consumers are often resistant to meat that exhibits visible IMF. Thus, several studies have demonstrated that the level of overall acceptability of pork increases with IMF content up to 2.5–3.5% [102, 104]. However, other studies observe that a significant number of consumers prefer less marbled pork (1 to 1.5% IMF) [100, 105]. A distinction between consumer groups based on the preference for moderately or slightly marbled beef has also been noted and associated with taste or nutritional expectations, respectively [106]. Thus, the assessment of relationships between IMF content and the sensory attributes of meat depends on the dietary habits and cultures of the consumers and on the considered products. For example, the tenderness, juiciness, and acceptability of dry ham have been demonstrated to increase with IMF content [107]. However, the reverse has been observed for cooked ham, whose acceptability decreases with an increase in IMF from 2 to 4% in the* Semimembranosus* muscle [108]. Similarly, a variation from 2.9 to 10.7% in IMF differently influences the acceptability of salmon fillets depending on the particular product. A decreased IMF content is more favorable for the baked fillet, whereas the opposite is true for smoked fillets [109].

## 7. Conclusion

The three main components of muscle (i.e., muscle fibers, connective tissue, and adipose tissue) are involved in the determination of various meat quality dimensions but to varying degrees depending on species, muscle type, and postslaughter meat-processing techniques. The relative independence among the characteristics of these three major muscle constituents suggests that it is possible to independently manipulate these characteristics by genetic, nutritional, and environmental in order to control the quality of products and thus better fulfill the expectations of producers, meat processors, and consumers. Therefore, precise knowledge regarding the structural and biochemical characteristics of each muscle component and their relationships with growth performance and meat quality dimensions is a prerequisite to understanding and controlling the biological basis of the quantity and quality of animal products. Future research should focus on the modulation of muscle properties that determine the major components of meat quality in the different species: tenderness in cattle, water-holding capacity and tenderness in pigs and poultry, and flesh texture in fish.

## Figures and Tables

**Table 1:** Biological characteristics of muscle fiber types^1^ [6].

	I	IIA	IIX	IIB
Contraction speed	+	+++	++++	+++++
Myofibrillar ATPase	+	+++	++++	+++++
Contraction threshold	+	+++	++++	+++++
Contraction time per day	+++++	++++	+++	+
Fatigue resistance	+++++	++++	++	+
Oxidative metabolism	+++++	++++	++	+
Glycolytic metabolism	+	++++	++++	+++++
Phosphocreatine	+	+++++	+++++	+++++
Glycogen	+	+++++	++++	+++++
Triglycerides	+++++	+++	+	+
Phospholipids	+++++	++++	+++	+
Vascularization	+++++	+++	+, ++	+
Myoglobin	+++++	++++	++	+
Buffering capacity	+	+++	+++++	+++++
Z line width	+++++	+++	+++	+
Diameter	++	+, ++	++++	+++++

^1^+: very low; ++: low; +++: medium; ++++: high; +++++: very high.
